# Contextualising ventilation decisions: an ethnographic study of factors shaping interprofessional decision-making

**DOI:** 10.1186/s12912-026-04381-w

**Published:** 2026-02-07

**Authors:** Andreas Küpper, Marcel Schmucker, Laura Hahn, Astrid Elsbernd, Simon Kitto, Cornelia Mahler

**Affiliations:** 1 Institute for Health Care and Nursing Sciences, Faculty of Social Work, Education and Nursing Sciences, Esslingen University of Applied Sciences, Esslingen, Germany; 2 Department of Nursing Science, University Hospital Tübingen, Tübingen, Germany; 3 Lee Kong Chian School of Medicine, Nanyang Technological University Singapore, Singapore, Singapore

**Keywords:** Interprofessional collaboration, Decision-making, Critical care, Contextual factors, Clinical ethnography, Respiratory support

## Abstract

**Background:**

Ventilation is a core domain of intensive care, and decisions about its management are frequent, complex, and involve multiple professions. While professional responsibilities in such decisions have been examined, little is known about the contextual factors that shape ventilation-related decision-making and the distribution of responsibilities among professional groups. This study aims to identify and analyse such factors.

**Methods:**

An ethnography was conducted in two German ICUs between December 2023 and July 2025. Data comprised 97 h of observation and 17 episodic interviews with nurses and physicians. Analysis followed the Qualitative Analysis Guide of Leuven (QUAGOL).

**Results:**

Ventilation-related decision work was shaped by a constellation of interdependent contextual factors. These factors were organised in an analytic heuristic structured by level (system, unit, actor) and dimension (structural, situational). In exploring five factors in greater depth – *experience*, *‘significant others’*, *rounds*, *‘bed shortage’*, and the *pandemic* – the analysis illustrates how contextual conditions interact across levels to shape both responsibilities and processes of decision-making in everyday ICU practice.

**Conclusions:**

Ventilation-related decision work in ICUs is embedded in and shaped by multiple interacting contextual conditions. By providing an analytic heuristic to organise and relate these factors, this study offers a structured perspective for understanding how context shapes ventilation-related decision work in everyday practice and provides a basis for further research, education, and reflective practice.

**Clinical trial number:**

Not applicable.

**Supplementary Information:**

The online version contains supplementary material available at 10.1186/s12912-026-04381-w.

## Background

Mechanical ventilation is a defining technology of intensive care [1, 2]. Decisions regarding ventilatory management are both frequent and highly complex [3–5], not least because they are interrelated with other aspects of care, such as sedation [5–7] or the use of mechanical restraint [8–10].

The provision of safe and high-quality intensive care is closely linked to effective interprofessional collaboration (IPC), which benefits both patient outcomes and staff well-being [11–15]. As ventilatory support routinely involves multiple professional groups [4, 16–20], IPC is particularly critical for ventilation-related decision-making, as it integrates perspectives and information from all relevant professionals [4, 21, 22].

IPC is shaped by relational, organisational, and situational conditions of everyday practice [23, 24]. The same applies to clinical decision-making, which is influenced by contextual factors such as care culture, available resources, and team [25–27].

Previous studies have provided valuable insights into decision-making processes related to ventilation, often focusing on specific professional groups or aspects of care [25, 26, 28–31]. Variations in decisional responsibilities across professions are also well described [16–20]. However, despite the central role of respiratory support in intensive care and the involvement of multiple professional groups, there remains limited understanding of the contextual factors that shape ventilation-related decision-making in everyday practice. The aim of this article is to identify and understand the contextual factors that shape interprofessional ventilation-related decision-making in ICUs and their interplay in everyday practice. Such an understanding may help explain variations in decisional responsibilities across units within the same healthcare system and provide a basis for reflecting on more targeted approaches to support interprofessional ventilation decision-making.

To address this aim, we draw on an interactionist perspective and conceptualise decision-making as work – the coordinated activities and interactions through which professionals align their contributions [32]. This lens allows decision-making to be examined not as an isolated cognitive act, but as an ongoing, socially situated accomplishment shaped through interaction. Such decision work unfolds within a critical illness trajectory, understood as an evolving process shaped by multiple actors rather than solely the patient’s course of illness [33]. From a pragmatist stance, we define context as empirically relevant conditions that shape decision-making practices without being part of those practices themselves [34]. To approach the empirical material with openness while sharpening our perspective, we used ‘interprofessional’ as a sensitising concept, referring broadly to interactions between members of different professional groups involved in decision work.

Our guiding research question is therefore: In which ways do contextual factors shape interprofessional ventilation-related decision work in ICUs?

## Methods

### Research approach and paradigm

This study was situated within an interpretive paradigm. Drawing on symbolic interactionism, this perspective assumes that social reality is continuously produced through interaction and the interpretation of situations [35]. Within this interpretive lens, we used ethnography to examine everyday practices and interactions in situ as a means of identifying the contextual factors that shape decision-making [36–38].

### Researcher characteristics and reflexivity

The research team comprised nursing scientists who are registered nurses (AK, MS, LH, AE, CM) and a medical sociologist (SK). Several team members have a research focus on Interdisciplinary Collaboration of Nursing, Medicine, and Technology (AK, MS, AE). AK, MS, and CM have prior clinical experience in intensive care. SK contributed expertise in ICU ethnography. In addition, both CM and SK brought substantial experience in interprofessional and qualitative research.

The first author’s (AK) clinical experience in intensive care facilitated access to the field and supported an in-depth understanding of everyday practices and professional interactions. Although he had not previously worked on the units under study, his professional affiliation with one of the included occupational groups may have shaped how access to participants was negotiated across professional groups. Reflexive considerations related to this positionality were addressed throughout the research process and are further elaborated in the section on rigour and trustworthiness.

### Ethical considerations

Ethical approval was obtained from the Ethics Committee of the University Hospital Tübingen (project number 142/2023B02). Written informed consent was collected from all interview participants. For participant observation, informed consent was obtained from the professionals being accompanied. Transparency was ensured through information sessions organised on both ICUs and the provision of written study information to staff prior to data collection, and staff were informed that participation was voluntary and could be withdrawn at any time.

### Context and setting

The study was conducted in two adult ICUs of a large German university hospital, providing high-acuity intensive care within a tertiary care setting. One unit was a surgical ICU led by anaesthesiology, had 40 beds and cared for and treated critically ill surgical patients, including all patients requiring extracorporeal membrane oxygenation (ECMO) or extracorporeal life support (ECLS). The second unit was a medical ICU with 21 beds, providing intensive care for a broad spectrum of critically ill patients from internal medicine and neurology. The unit delivered comprehensive intensive care support, including advanced organ support, with the exception of extracorporeal life support, which was managed on the surgical ICU. Direct patient care on both units was provided by interprofessional teams, including registered nurses with and without specialist intensive care training, physicians from different medical specialties, and allied health professionals such as physiotherapists, occupational therapists, and speech and language therapists. At the time of data collection, no respiratory therapists were employed on either ICU.

### Sampling and participants

Participant selection followed a purposive sampling strategy, focusing on nurses and physicians who, based on prior research [4, 14, 20] and the researchers’ professional experience in German ICUs, are central actors in interprofessional clinical decision work. While observations encompassed a wider range of professional groups involved in everyday intensive care, interviews and job shadowing were limited to nurses and physicians. To obtain a broad picture, we applied maximum variation and stratified sampling [35] with regard to hierarchical position and professional experience, which early observations indicated as relevant factors shaping decision work. As part of an iterative process consistent with theoretical sampling [39], the study aim and sampling strategy evolved together. While the initial focus was on clinical decision-making in general, ongoing data generation and analysis led to a more specific emphasis on ventilation-related decisions.

### Data collection and processing

Participant observation combined with in-field ethnographic interviews [36, 40], were the primary method of data generation. For participant observation, nurses and physicians were approached by the first author. For interviews, participants from the same occupational groups were initially invited through written interview invitations and information material on the units; during later stages of fieldwork, potential interviewees were approached directly on the units by the first author. In total, 97 h of observation were conducted between December 2023 and July 2025 by the first author. Early observations involved shadowing across shifts to gain a broad understanding of routines and structures. Later, the focus shifted to situations that were especially relevant for the emerging analysis. Ethnographic field interviews with staff were conducted alongside observations and integrated into the fieldnotes, which were subsequently expanded into detailed protocols immediately after each session [36, 41]. Additionally 17 episodic interviews were conducted by the first author between April and July 2025 with nurses (*n* = 12) and physicians (*n* = 5). These interviews complemented the fieldwork by providing access to the semantic and narrative knowledge of both groups [35, 42].

The interview guide was specifically developed for this study by the first author, informed by Flick’s approach to episodic interviews [42], the current state of the analysis and refined within the research team. The interview guide was piloted with a nurse, a physician, and a nurse who had completed additional training in respiratory therapy, each with ICU experience. An English version of the guide is available in Supplementary Material 1. These planned interviews were conducted face-to-face or via Webex, recorded, and transcribed verbatim using AI-assisted software aTrain [43]. All transcripts were checked and corrected by the first author.

Data collection was conducted in an iterative manner, alternating between data gathering and analysis. Insights from earlier stages informed the subsequent fieldwork and the interviews. Observations were continued until ongoing analysis indicated redundancy in the identification of decision-relevant situations and contextual factors, signalling theoretical saturation and thereby marking the endpoint of both observational and interview data collection [36, 41, 44].

### Data analysis

Data analysis was guided by the Qualitative Analysis Guide of Leuven (QUAGOL), an analytic framework that builds on heuristic principles of Grounded Theory, including iterative analysis and constant comparison [45]. These principles have been shown to align well with ethnographic approaches, as both emphasise contextualised, case-oriented analysis and the systematic comparison of empirical situations [46, 47]. A particular strength of QUAGOL is that it provides a structured approach to developing an in-depth understanding of cases prior to formal coding [48].

As QUAGOL was originally developed for interview data, the approach was adapted in this study to incorporate ethnographic observation protocols. Observational data were first reconstructed into narrative, case-oriented descriptions that captured decision-relevant situations and interactions. These reconstructions enabled the application of QUAGOL’s core analytic steps, including in-depth familiarisation, conceptual scheme development, and iterative coding [45]. Analysis proceeded through continuous movement between individual cases and cross-case comparison, following the logic of constant comparison to examine similarities and differences across decision situations and settings [45, 48].

This approach allowed us to integrate the depth of ethnographic data with a systematic analytic structure, supporting the identification and refinement of contextual factors shaping ventilation-related decision-making. Coding and data management were subsequently supported by the software MAXQDA [49].

### Rigour and trustworthiness

Rigour, guided by ethnographic criteria [50], was pursued through diverse interrelated strategies. Prolonged fieldwork and triangulation of observations and different types of interviews supported a credible account of lived realities. Reflexivity was cultivated through reflexive memos by the first author (AK) [44], peer debriefings with team members (AK, MS, LH), and feedback loops with senior qualitative researchers (CM, SK, AE). Interdisciplinary workshops with nurses, physicians, and other professionals were visited to discuss the material. During periods in which team-based interpretation was not feasible, the first author used interpretive dialogues with ChatGPT as a reflexive support tool. No empirical data were entered into the system. Instead, provisional concepts and analytic questions were used to stimulate critical reflection and challenge emerging assumptions. ChatGPT outputs were not treated as analytic results but served solely to support reflexive deliberation; all analytic decisions and interpretations remained the responsibility of the research team. Finally, elaboration of selected factors aimed to strengthen the study’s contribution to critical care research and practice by opening up avenues for further reflection and discussion.

## Findings

The following findings present an analytic account of the contextual conditions that shaped ventilation-related decision work in everyday ICU practice.

### A heuristic of contextual factors in ventilation-related decision work

Data analysis revealed that ventilation-related decision work in intensive care was embedded in and shaped by a constellation of contextual factors. These factors were heterogeneous in nature and could not be meaningfully understood in isolation. Rather, they interacted across different levels and situations, shaping how ventilation-related decisions were negotiated and enacted in everyday practice.

**Fig. 1:**
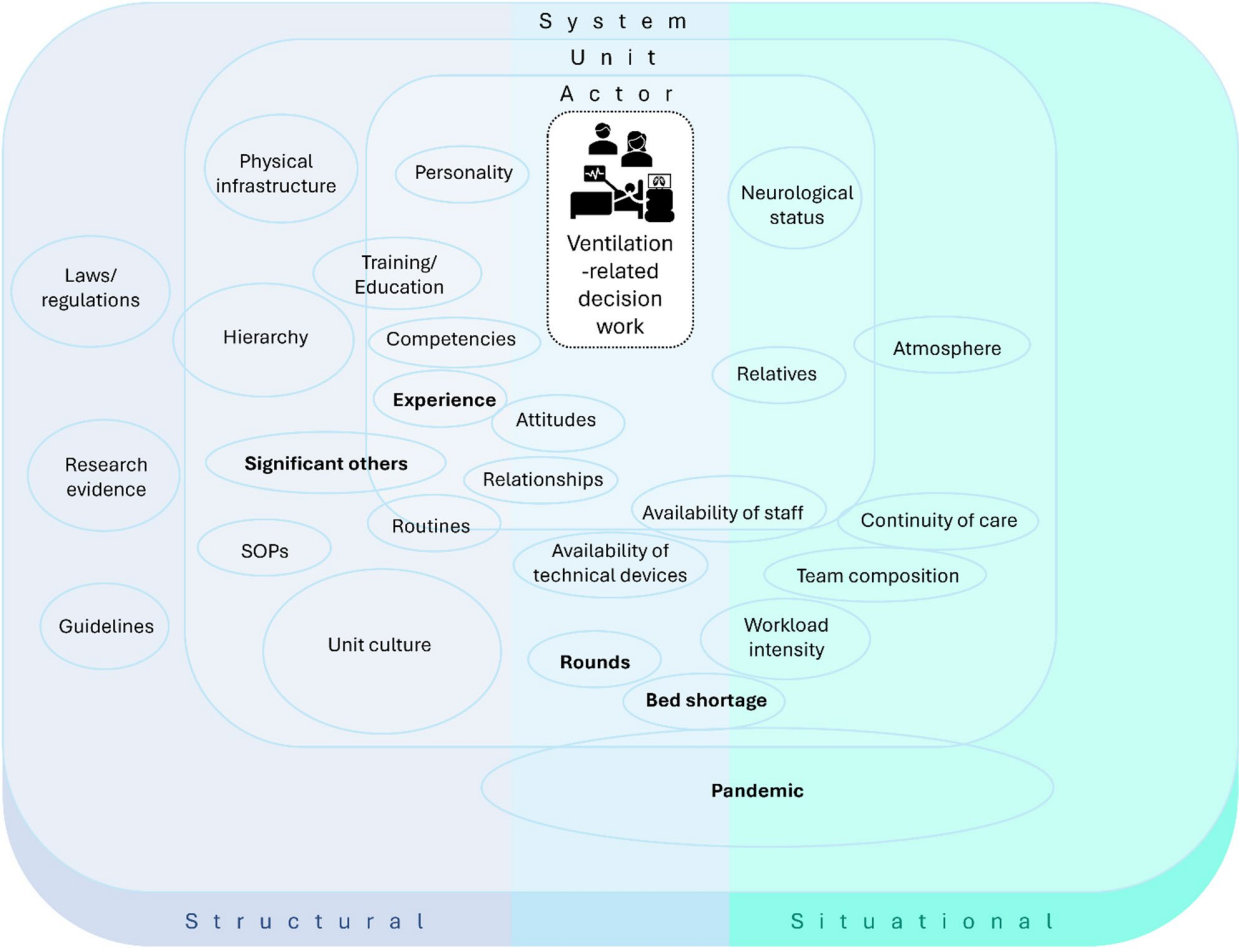
Analytic heuristic of contextual factors shaping ventilation-related decision work

To organise these findings, we inductively developed an analytic heuristic, presented in Fig. 1. The heuristic situates ventilation-related decision work at the centre and visualises how contextual factors operating at the system, unit, and actor levels surround and shape this work. The positioning of factors is not intended as precise or exhaustive, but serves as an analytic aid to reflect on how different constellations of conditions become relevant in specific decision situations.

The system level captures broader institutional and regulatory conditions, such as legal frameworks, guidelines, and research evidence, as well as extraordinary system-wide influences such as the COVID-19 pandemic. The unit level refers to organisational arrangements and local practices, including routines, rounds, unit culture, and hierarchical structures. The actor level encompasses individuals as situated actors, including relatively stable attributes such as experience, competencies, training, and personal characteristics, as well as situational conditions that directly shape decision work, such as patients’ neurological status or the availability of technical devices. Individual characteristics were not treated as explanatory variables, but as contextual conditions whose relevance became visible in the specific configuration of ventilation-related decision work.

Across these levels, contextual factors differed in how they shaped decision work. Some constituted underlying structural conditions that provided a relatively stable framework within which decision work unfolded, while others emerged in specific situational constellations and became relevant only in particular moments. Several factors operated at the intersection of structure and situation, highlighting the permeability of analytic boundaries and underscoring the heuristic rather than classificatory character of the model.

From the broader set of identified contextual conditions, five factors were selected for closer examination in the following sections. These factors were chosen because they exemplify different levels and modes through which context shaped ventilation-related decision work. *Experience* was selected as it was mentioned in every episodic interview and repeatedly observed in practice, illustrating how individual attributes shape participation and positioning in decision work. ‘*Significant others’* capture how particular individuals exert influence within units and exemplify the permeability of professional boundaries in ventilation-related practice. *Rounds* were chosen as a central arena of interprofessional collaboration, highlighting how organisational structures and everyday practices intersect. The factor *‘bed shortage’* demonstrates how system- and unit-level demands are translated into situational pressures that directly reshape ventilation-related decisions. Finally, the *pandemic* was included as a factor illustrating how extraordinary circumstances can disrupt established routines, open spaces for negotiation, and lead to transformations in practice whose effects extend beyond the acute crisis itself.

All additional factors found in the heuristic (see Fig. 1) are concisely described in Supplementary Material 2. For readability, not every analytical statement is accompanied by a verbatim quote; identifiers such as *[E1]* or *[SO2]* link to the data in Supplementary Material 3, while key excerpts are presented in the text. All quotations and excerpts originally in German were translated into English by the first author (AK), with support from ChatGPT and DeepL, for presentation in the manuscript and supplementary materials.

### Experience

*Experience* was cited in all interviews as a factor shaping ventilation-related decision work among both nurses and physicians. Experience was not predefined analytically but followed the meaning attributed to it in the field, where it referred to practice-based familiarity with ICU work. A common pattern was that clinicians with limited ICU experience tended to involve others with greater expertise, particularly in relation to ventilation. In this case, experienced nurses were frequently identified by both professional groups as primary contact persons [see Supplementary Material 3 - E1].

Experience was also described as shaping (non-)interaction across professional groups. Participants reported that ventilation or sedation settings were more often changed by physicians without informing less experienced nurses [E2]. It was further described that physicians responded less promptly to concerns voiced by junior nurses, for example when they raised concerns about failing non-invasive ventilation.

*Well*,* what happens is that we go to the doctor and say*,* hey*,* this is getting critical*,* think about it (…). And then*,* I have to say this as a point of criticism*,* it is quite decisive whether it is a less experienced nurse who seeks help from the physicians or someone experienced. With the less experienced ones – although I do not understand this at all – the doctors come later. (Interview 14*,* nurse*,* extensive ICU experience)*

Experienced clinicians were more likely to move beyond prescribed standards [E3] or contradictory physicians’ orders, drawing on their own judgement to advance decisions more swiftly towards implementation [E4].

By contrast, less experienced clinicians, whether physician or nurse, were reported to rely more heavily and more frequently on measurable parameters, such as blood gas results, when forming their judgement [E5].

Experience was further described as a relevant factor in enforcing positions during the negotiation of decisions [E6]. Charge nurses, drawing on their seniority, sometimes assumed an advocacy role for less experienced colleagues – for instance by taking up their concerns about intubation and voicing them with physicians or senior physicians [E7].

Overall, experience shaped ventilation-related decision work in multiple ways, establishing it as an omnipresent contextual factor.

### Significant others

Borrowing terminology from symbolic interactionism, our analysis identified *‘significant others’* as a contextual factor in ventilation-related decision work. Originally significant others are those concrete reference persons whose perspectives are internalised and thereby contribute decisively to the development of the self [51]. As applied in this study, the term refers to individuals who exert influence on clinicians’ ventilation-related decision work across professions, even when they are not physically present, thereby shaping elements of unit culture related to ventilation practices.

One recurring group of ‘significant others’ were senior consultants, whose preferences in ventilation management shaped daily practice:

*I ask the nurse why these ventilation modes were chosen. She explains that this is due to a consultant’s preference*,* which*,* from a pulmonology perspective*,* is considered better for pulmonary integrity. (Observation 12)*

This group of ‘significant others’ also had a marked influence on how rounds were structured, thereby affecting interprofessional decision work on ventilation more broadly [SO1].

The data further pointed towards the influence of clinicians involved in onboarding new team members. This influence manifested both in workshop formats, which provided newcomers with essential competencies in ventilation support, and in the day-to-day accompaniment of new staff during their onboarding phase on the unit. Importantly, their impact extended beyond the period of direct onboarding, as the norms and practices they conveyed continued to shape how newcomers approached ventilation-related decision work [SO2].

Interestingly, the data also revealed ‘significant others’ whose influence was attributed primarily to their widely acknowledged expertise and engagement with ventilation-related issues [SO3] — even when they also held hierarchical positions or were involved in onboarding. This recognition, while crossing professional boundaries [SO4], remained specific to the unit.

*In the past we had a very good respiratory therapist*,* [name]. He had quite an important role on the unit. (…) That was an absolute stroke of luck for our unit. (…) These are individuals who*,* especially the pioneers*,* sometimes bring in far above-average engagement. And he also had many years of experience as an intensive care nurse. And*,* well*,* he almost had the status of a consultant here. He was one of my closest confidants on the unit. (Interview 3*,* consultant*,* extensive ICU experience)*

In summary, ‘significant others’ were involved in decision work not necessarily through their physical presence, but by the ways in which their positions and routines were taken up and regarded by others.

### Rounds

*Rounds* were enacted in our material as organisationally fixed situations that structured how different professional groups became involved in ventilation-related decision work.

We observed different formats, which both reflected and contributed to unit-specific patterns of participation. On one ICU, monoprofessional rounds, which also included the physicians’ shift handover, took place at the central workstation and were strongly supported by IT infrastructure. These rounds typically involved gathering and discussing information among physicians, drawing on a wide range of information sources, and also functioned as an arena for medical training. The decisions usually concerned overarching questions of ventilatory management rather than adjustments of specific parameters.

In this mode, nursing staff became involved only afterwards during the subsequent bedside round. It was observed that some nurses adopted a more passive stance, whilst others participated actively, seeking to put forward their perspective [R1].

The weaning round, held weekly on both wards, was the most distinctly interprofessional format observed. It brought together physiotherapists, speech and language therapists, occupational therapists, social workers, nurses, and physicians, with each discipline contributing its perspective [R2]. On one ICU the round was nurse-led and conducted at the bedside, while on the other it was physician-led and took place in a separate room. The round was explicitly oriented towards ventilation-related decision work and often embedded the patient’s weaning situation in a broader clinical and social context.

Staff perceptions of its usefulness varied considerably, and these differences seemed to shape how actively individuals engaged with it [R3].

In sum, rounds proved to be a relevant contextual factor in interprofessional ventilation-related decision work, yet the way they shaped this work was contingent on how they were staged in practice and how participants chose to take part in interprofessional decision work.

### Bed shortage

The factor is labelled *‘bed shortage’*, reflecting the field’s own language. Yet, what was actually observed was a shortage of nursing staff: both units had vacant beds that could not be run due to limited nursing staff resources.

When the number of patients on the unit exceeded what could be safely managed under the usual nurse-to-patient ratio, or when new admissions were announced despite staff already being fully allocated, ventilation-related decision work became noticeably compressed. In such situations, the involvement of others was reduced, discussions were sometimes bypassed or became more contentious [BS1], and even far-reaching decisions were often implemented immediately or required explicit justification if delayed [BS2]. Periods of ‘bed shortage’ thus shifted the weighing of considerations from the clinical towards the organisational:

*Under the pressure of a ‘bed shortage’*,* a consultant expresses frustration that a patient has not yet been extubated*,* referring to ventilation parameters in the electronic documentation. (…)*

*Later*,* I learn that the consultant extubated the patient without prior consultation with another consultant*,* who subsequently reacted with irritation. (Observation 4)*

Overall, ‘bed shortage’ was observed only occasionally, but when present it strongly shaped the mode of ventilation-related decision work.

### Pandemic

In our analysis, the COVID-19 *pandemic* was conceptualised as a system-level contextual factor that continued to shape ventilation-related decision work during the period of data collection. Decisions made during the active phases of the pandemic were considered particularly challenging, both because of the severity of pulmonary disease and because of limited routine in handling such cases, which also affected the extent of interprofessional involvement [P1].

While these accounts referred primarily to experiences during the acute phases of the pandemic, other influences of COVID-19 were described as having a more enduring impact on ventilation-related decision work and remained observable during the period of data collection. In particular, the pandemic was portrayed as disruptive, as it created space for new practices [see SO4] while previously taken-for-granted routines no longer held:

*(…) I once learned that this is an NIV failure. I believe that during COVID this all more or less dissolved into thin air*,* and patients were kept on NIV much longer. (Interview 1*,* nurse*,* mid-level ICU experience)*

In addition, the pandemic period was described as a time of growth in ventilation-related competencies, and new forms of knowledge management in relation to ventilation were established [P2].

Overall, the pandemic functioned as a situational backdrop that not only challenged established routines but also fostered competence growth and new ways of managing knowledge, thereby leaving a lasting imprint on ventilation-related decision work.

The heuristic (Fig. 1) integrates the five factors presented here into an analytic perspective on how contextual configurations shape ventilation-related decision work.

## Discussion

This study asked in which ways contextual factors shape interprofessional ventilation-related decision work in ICUs. Drawing on ethnographic fieldwork in two German ICUs, it shows that such decision work is shaped not by single contextual factors, but by dynamic constellations of interdependent structural and situational conditions operating across system, unit, and actor levels.

### Levels of contextual factors and their interrelations

The influence of individuals, their composition [24], and particularly professional experience [52–55], on situated decision work is well documented in studies on interprofessional practice [56] and clinical decision-making [52, 53] in intensive care. Our findings extend this literature by making this influence explicit in relation to ventilation-related decision work.

However, our analysis shows that these individual influences cannot be understood in isolation, but are embedded in and shaped by contextual conditions operating at other levels.

Sensitising for this complex interweaving is one of the central contributions of this study and a strength of the analytical heuristic presented in Fig. 1.

The interplay across levels can be illustrated particularly well by the factor ‘bed shortage’: In the German ICU context studied here, the apparent lack of beds primarily reflects a shortage of nursing staff [57, 58], further aggravated by the COVID-19 pandemic - another contextual factor in our material. Through the factor of ‘bed shortage’, these systemic conditions exert their influence at the actor level by altering individual behaviours and compressing interprofessional decision-making processes.

### Unit culture on ventilation decision work as a negotiated order

The ICU-specific culture on ventilation-related decision work – put simply, who is expected and allowed to do what and in what way – again reflects the interplay of contextual factors and offers an explanation for variations in decisional responsibilities within health systems [16–20]. This perspective is consistent with and extends earlier publications that have taken unit culture into account [25, 59].

Such culture can be seen as a form of social order at unit level. Following the concept of negotiated order, it is not fixed but the result of continuous negotiations in daily practice – though bounded by limits. Negotiated order highlights that organisational arrangements and professional boundaries are not given, but constantly reproduced and adjusted through interaction [60]. Several of the contextual factors identified in this study seem to shape these negotiations and at the same time influence the boundaries within which they take place.

For example, ‘significant others’ – clinicians who, by virtue of their position, expertise, or commitment, exert considerable influence – appear to play a central role in negotiating the order. At the same time, they depend on the existing order to exercise this influence. The introduction of a new ventilation mode by a highly valued nurse illustrates this: it extended the accepted options in ventilation, yet required a unit culture that allowed nurses to act in a domain traditionally reserved for physicians. At the same time, our findings resonate with literature on ‘non-negotiables’ in clinical practice, showing that factors such as ward round structures or hierarchical routines are often experienced as fixed [54, 55, 61, 62].

Thus, negotiated order always operates within limits, grounded in system-level conditions like laws and regulations, but sometimes also set by ‘significant others’ – most often senior physicians – for instance by insisting on a specific ward round structure.

### Intra and interprofessional interaction

Previous studies show that ventilation-related decision-making involves multiple professional groups [4, 16, 20], a pattern reflected in our findings through contextual factors enacted in intra- and interprofessional interaction. Rounds, for example, can serve as a central context for interprofessional interaction [63] – including in ventilation-related decision work, as our findings demonstrate. Consistent with prior research the extent to which the observed ward rounds were able to realize this potential for IPC depended on additional contextual factors, such as the way the rounds were structured and attitudes of the actors involved [64, 65].

In rounds, but also in other moments of inter- and intra-professional interaction, hierarchies showed to be relevant contextual factors shaping ventilation-related decision work, with senior medical staff usually taking a dominant role. This finding has been repeatedly demonstrated in international ethnographic research [54, 66–69].

However, the extent of this dominance varied depending on other context factors such as the constellation of actors present and their individual characteristics.

Interprofessional frictions in ventilation-related decision work appeared to arise mainly when residents rotating onto the unit were not yet familiar with the unit culture, or when formal hierarchical authority and experiential expertise were misaligned—for example, when less experienced physicians issued instructions to more experienced nurses. In such situations, this tension manifested both as overt resistance, such as withholding cooperation, and as more subtle forms of compliance accompanied by internal dissent. These situations, in turn, reflected broader contextual factors such as team composition, more specifically a combination of hierarchy and professional experience.

Overall, our findings show that ventilation-related decision work is shaped by a complex web of interdependent contextual factors with different characteristics. At the same time, decision work is embedded in a negotiated order – both the outcome of these interwoven factors and, simultaneously, a contextual factor in its own right. This perspective provides a basis for further research and for reflective engagement in ICU practice by highlighting how decision work is continuously shaped and constrained by its context.

### Methodological reflections and limitations

In reflecting methodological issues and discussing the limitations of this study, we draw on Richardson’s five criteria for evaluating ethnographic research: substantive contribution, aesthetic merit, reflexivity, impact, and expression of reality [50].

As an ethnographic study, our analysis is grounded in the specific characteristics of the setting: two ICUs in a German university hospital, situated within a nursing system strongly rooted in vocational education. This contextual embeddedness is integral to the design, as ethnography seeks to make the local visible in order to generate conceptual insights. At the same time, previous studies suggest that questions of decisional responsibility in ventilation do not differ fundamentally between Germany and other countries [20], indicating that the concepts developed here may be of broader relevance.

In terms of aesthetic merit, condensing data for readability inevitably reduced some descriptive richness. Illustrative material is therefore provided in the supplement. As the original data were generated in German, nuances may have been lost in translation. As the first author is not a native speaker, support from co-authors and AI tools helped to ensure linguistic clarity.

The first author’s background as a former ICU nurse was treated as an analytic resource that facilitated field access and contextual understanding, while requiring ongoing reflexive engagement. Measures to support reflexivity are described in the section on rigour and trustworthiness.

The study identified a broad range of factors but could not examine each in depth, leading to a necessarily selective treatment. Our approach was to elaborate exemplary factors and embed them in the analytical heuristic. The impact lies less in providing definitive answers than in stimulating further questions and offering perspectives that help understanding one facet of everyday ICU practice.

In terms of representing practice, the article offers only a partial account. The analysis focused on contextual factors, while the processes of decision work themselves are not presented. This intentional fragmentation was chosen to reduce complexity. Recruitment challenges resulted in differing numbers of interviews between nurses and physicians, which may appear imbalanced. However, for our ethnographic and interpretive approach, equal distribution was not required, as the aim was not representativeness but conceptual depth. Other professional groups were scarcely represented; while we acknowledge their relevance for patient care [70], our data suggest that in the specific settings observed decision work was primarily shaped by nurses and physicians.

A further limitation is the near absence of the patient. This reflects not only our study design but also a broader empirical reality: the silencing of patient voices in ICU practice, especially under mechanical ventilation [71]. Yet, as Sterr et al. [72] show, sedated or ventilated patients retain perceptions of agency and vulnerability, underscoring the importance of including their roles. At the same time, this absence is partly rooted in our analytic focus: the patient and their condition were treated as constitutive elements of the decision process itself [52] rather than as contextual factors. Future publications aim to address both the patients’ role and the process of decision work more explicitly.

## Conclusion

We conceptualised ventilation-related decision work as embedded within the critical illness trajectory and examined the contextual conditions under which this work takes place. Our findings show that ventilation-related decision work is shaped by a complex interplay of interdependent contextual factors that influence one another and become relevant in specific constellations. Decision work thus appears as situated within organisational, professional, and material environments.

The main contribution of this study lies in the empirically driven identification and analytic organisation of contextual factors relevant to interprofessional decision work in ICUs. By arranging these factors within a heuristic and illustrating their interrelations, we offer a structured way to reflect on the conditions under which ventilation-related decisions are negotiated and enacted. For research, this framework opens up questions about how different contextual constellations shape decision work. For education and practice, it provides a tool to support reflection on how local circumstances influence everyday decision-making.

## Supplementary Information

Below is the link to the electronic supplementary material.

Supplementary Material 1

Supplementary Material 2

Supplementary Material 3

## Data Availability

The data supporting the findings of this study are available from the corresponding author upon reasonable request. Due to privacy restrictions, access to the full data is limited. However, additional excerpts from the data are provided in Supplementary Material 2 and 3.

## Declarations

### Declaration of AI use

During the preparation of this manuscript, generative AI tools were employed at several stages. aTrain was used for transcription of interview data. DeepL and ChatGPT supported the translation of data excerpts from German to English. ChatGPT was further used as a writing assistant to improve clarity, grammar, and style, and as an interpretive partner to simulate multidisciplinary group discussions during data analysis. No empirical data were shared with the AI; instead, the first author presented his own analytic considerations to this simulated group and used the responses to critically reflect on his reasoning, challenge his perspective, and generate new impulses. The authors reviewed and edited all AI-assisted content and take full responsibility for the final version of the manuscript.

## Abbreviations

ECLS: Extracorporeal life support
ECMO: Extracorporeal membrane oxygenation
ICU: Intensive care unit
IPC: Interprofessional collaboration
QUAGOL: Qualitative Analysis Guide of Leuven
SOP: Standard operating procedure
