# Supplementary material for: Contextualising ventilation decisions: an ethnographic study of factors shaping interprofessional decision-making
Source: BMC Nurs. 2026 Feb 7;25:237. doi: 10.1186/s12912-026-04381-w (PMC12994234; doi:10.1186/s12912-026-04381-w)
Supplement: Supplementary file 1 — Supplementary Material 1 [file 12912_2026_4381_MOESM1_ESM.docx]

**Episodic interview guide**

**Guiding research questions:**

- How do nurses and physicians negotiate ventilation-related clinical decisions in intensive care units?
- ow do technical devices shape interprofessional clinical decision-making in intensive care units?
- How do technical devices shape the professional boundaries between nurses and physicians in intensive care units?

| **Introduction** |
| --- |
| Thank you for taking the time to speak with me today. As you may know, I am particularly interested in how clinical decisions—such as those concerning ventilation—are made in the intensive care unit. As you are likely aware, I have already conducted participant observation on your unit. Today, however, I am not seeking to verify my observations, but rather to learn about your personal perspective and experiences. In this interview, I will ask you various questions on the topic and repeatedly encourage you to describe concrete situations that fit. For my research, it is important that you describe the examples and your related thoughts in great detail. There are no right or wrong answers. |

| *#* | *Main question/Narrative stimulus* | *Follow-up or probing question* |
| --- | --- | --- |
|  | When you think of a typical workday in your unit: when do decisions about patient ventilation occur? How does this usually proceed? Can you describe a real example? | - Are there other types of ventilation-related decisions? What do they look like? Can you give an example? - Who is usually involved in such decisions? What determines who is included? Are there situations where certain people are intentionally not involved? Why are they excluded? - What happens when a decision is finalized? Who makes that decision? - How are the decisions then implemented? Who is responsible for this? How is it communicated? - How would you describe the collaboration between nurses and physicians in such decision-making processes? |
|  | What does your own position on a ventilation decision depend on?  What influences your attitude in such situations? | - Which arguments or pieces of information do you find particularly convincing or important? Which have little influence on your attitude toward a decision? - How important is the opinion of others? Which people influence your own opinion the most? - What role do devices such as the ventilator, monitor, or infusion technology play in this? Can you illustrate this with a personal experience? |
|  | When you think about the topic of ventilation as a whole, what tasks fall within your area of responsibility? | - Does this apply to all nurses/physicians? If there are differences, what do they depend on? - How would you ideally define your area of responsibility regarding ventilation? What would need to change for that to happen? |
|  | How would you describe your overall influence on ventilation decisions? Can you illustrate this with real situations? | - What hinders your influence on a decision? What promotes it? - If a decision is of particular importance to you, how can you increase your influence on it? Can you think of a concrete example? - Suppose a decision was made over your head or entirely against your own position, without your presence. How do you respond in such a case? |
|  | Let’s return to the technical devices. Taking the ventilator as an example: how do you see the roles of the different professional groups? Can you describe a situation that illustrates this? | - Apart from professional affiliation, what else determines who takes which role in handling technical devices? - Are there situations where technical devices promote collaboration between professional groups? What does that look like? - Are there situations where technical devices lead to conflicts? What kinds of conflicts are these? |
|  | Depending on the professional group, there are different tasks and responsibilities. I’m interested in whether and when these typical boundaries become blurred. Can you recall an experience in which this usual division was altered? | - Are there tasks or areas where responsibilities are absolutely clear and not open to discussion? - When does crossing these boundaries have consequences? What do those consequences look like? |
|  | Is there anything else you would like to share on this topic? | |
| Thank you very much for your participation. Do you have any questions for me? | | |
