# Supplementary material for: Contextualising ventilation decisions: an ethnographic study of factors shaping interprofessional decision-making
Source: BMC Nurs. 2026 Feb 7;25:237. doi: 10.1186/s12912-026-04381-w (PMC12994234; doi:10.1186/s12912-026-04381-w)
Supplement: Supplementary file 2 — Supplementary Material 2 [file 12912_2026_4381_MOESM2_ESM.docx]

**Supplementary Material 2: Explanation and linkage of contextual factors**

| **Actor level; structural** | |
| --- | --- |
| **Attitudes** |  |
| Clinicians’ attitudes towards collaborative decision-making appeared to shape both their willingness and the way they exchanged views with colleagues. | “In general, as a kind of final plea, I would wish that we sat together more often to really think about what we do, what we do and how we do it – and then to carry this through consistently, over an hour, a shift or even a whole day – and to evaluate afterwards whether it has actually made a difference or not.” *(Interview 4, nurse, extensive ICU experience)* |
| **Competencies** |  |
| Clinicians’ competencies, particularly regarding respiratory support, indicated influence on decision work by shaping awareness of treatment options and the ability to judge their value and implement them. | “But real expertise, I would say, especially [name of nurse] or [name of another nurse] – I think they are just so deeply into it and can explain things in such a complex way, or bring such a different perspective, (…).” *(Interview 9, nurse, early ICU experience)* |
| **Experience** | see article |
| **Personality** |  |
| Clinicians’ personalities showed to shape how their perspectives entered into decision work. More reserved individuals tended to voice their views differently than those with a more dominant presence. | A young male resident is speaking during the full-team handover from the early to the late shift, when collective attention shifts to a recently arrived external doctor in a white coat. A female resident, standing next to him, signals that he should step aside and remain quiet for a moment. I recall a nurse’s description of this male resident as “quiet, but competent.” In fact, he participates less in the usual negotiations during the handover than most of the other residents – even when it concerns patients under his care. *(Observation 5)* |
| **Relationships** |  |
| The relationships between clinicians seemed to influence how far they could engage with and take on one another’s perspectives during decision-making. | “I think especially in decisions like these, a lot depends on trust. In such a field, where very serious decisions are being made, it makes a big difference whether I have known someone for many years – in which case I can make such decisions differently – or whether I have only just got to know them.” *(Interview 5, nurse, extensive ICU experience)* |
| **Routines** |  |
| Routines showed influence on ventilation-related decision work, as clinicians tended to follow familiar paths when making such decisions. | “With ventilation, well … it’s sometimes difficult to move beyond the modes you are used to, even when there is evidence for it – for example, APRV in patients with ARDS.” *(Interview 13, resident, mid-level ICU experience)* |
| **Training/Education** | |
| Clinicians’ formal qualifications, such as specialist medical training or advanced nursing education, seemed to influence their inclusion in decision-making and the degree of autonomy they exercised. For instance, residents with specialist certification were more readily involved, while nurses with advanced training acted with greater independence. | “I would say the key factor in this decision-making is clearly the specialist training – the advanced training in critical care nursing. I would argue that colleagues who have completed it make these decisions much more independently.” *(Interview 5, nurse, extensive ICU experience)* |
| **Actor level; situational** | |
| **Availability of clinicians** |  |
| The availability of clinicians seemed to shape not only who was involved but also the scope of decisions that could be made at a given moment, for example when residents were required to take decisions they would not normally make alone because senior physicians were not yet available. | “Well, sometimes it’s difficult … for example, I was on the night shift and the patient’s respiratory status worsened significantly, with FiO₂ at 80 percent on NIV and PJP pneumonia. It was hard for me to make the decision and say, yes, this patient now needs to be intubated. In the end I did it, because it had already been defined by the consultants beforehand that if FiO₂ was 80 or higher on NIV, then intubation should follow.” *(Interview 7, resident, early ICU experience)* |
| **Availability of technical devices** |  |
| The technical devices at hand seemed to shape decision work by defining which modes of ventilation could be applied and how detailed monitoring was possible. Their characteristics not only constrained or expanded therapeutic options but also prompted exchange among clinicians, depending on who was familiar with a given device. | “And on this unit I was completely overwhelmed again by the whole philosophy of ventilation, because our machine, I think, can do quite a lot. We work extensively with the values it provides. (…) And the ventilation settings we can choose in our modes are much more detailed than elsewhere. During the induction or the equipment training by [name of expert nurse], I sat there again thinking, okay, I have no idea about ventilation. That’s how it feels.” *(Interview 1, nurse, mid-level ICU experience)* |
| **Neurological status** | |
| The patient’s neurological status seemed to determine how far they could take part in decision work and thereby influenced the process. | During the morning round, an awake patient with a chronic lung condition is directly addressed about his pulmonary situation. While the monitor shows an oxygen saturation of 93, the consultant asks: “Do you know what your oxygen saturation is at home?” The patient replies: “Yes, it’s about 95 to 98 at home.” The consultant comments that the lungs still need to improve and recommends increased mobilisation. *(Observation 13)* |
| **Relatives** |  |
| Relatives seemed to influence decision work, for example when their expressed wishes, referring to the presumed will of the patient, shaped the course of ventilation-related decisions. | “That’s something we deal with a lot. For example, with a multimorbid patient from a nursing home, we as nurses sometimes ask, ‘does this patient really need to be intubated?’ And then what often happens is a conversation with the relatives – ideally with the patient as well, depending on orientation or condition. And if the relatives say, we want ventilation, we want intubation, then that is what happens.” *(Interview 5, nurse, extensive ICU experience)* |
| **Unit level; structural** | |
| **Hierarchy** |  |
| Hierarchy was identified as a factor that strongly shaped decision work, especially regarding whose perspective ultimately prevailed. | “And when it comes to intensive care matters, there is room for us to raise objections or make suggestions. But at least when the senior consultants are present, we do not make the decisions. Still, we are heard, and if you have a good idea, it is accepted and implemented.” *(Interview 17, resident, early ICU experience)* |
| **Physical infrastructure** |  |
| Physical conditions such as room layout or transport facilities appeared to influence decision work, for example by framing what equipment could be used during patient transfers. | We enter the room of the responsible nurse, heading towards the bed from which the CT transfer is to take place. The question arises whether the bariatric bed can be transported in the lift or whether the patient has to be moved to a standard bed. It also has to be considered whether to take the small portable ventilator, with only limited settings, instead of the modern device integrated into the larger transport unit. *(Observation 15)* |
| **Rounds** | see article |
| **Significant others** | see article |
| **SOPs** |  |
| Standard operating procedures (SOPs) seemed to influence decision work by defining what was considered routine in ventilatory support and by making deviations visible, which in turn affected how clinicians engaged with decisions. | “That was explicitly written down again, because it was outside the norm. The SOP actually specifies, for example, when you have NIV failure or when you have to stop weaning – all of that is laid out explicitly in the SOPs. But if something is outside the norm, then it is explicitly documented.” *(Interview 10, nurse, early ICU experience)* |
| **Unit culture** | |
| Different ICUs appear to display distinct cultures of ventilation management and interprofessional collaboration, which shape the roles and degree of autonomy of the various professional groups involved. | “What I have experienced on the different ICUs I have worked on is that each unit has its own philosophy. (…) And in this way, you gather experiences across different units. But what I also encountered were ICUs where nursing staff were not involved at all in changes to the ventilation settings.” (Interview 1, nurse, mid-level ICU experience) |
| **Unit level; situational** | |
| **Atmosphere** |  |
| The atmosphere on the ward appeared to affect how clinicians interacted, including across professional groups, with implications for decision work. For example, humorous remarks during handover or the general mood—whether relaxed or tense—shaped how decisions unfolded. | On the way to the patient’s room for the more detailed handover, I already notice the relaxed atmosphere on the ward. It is entirely different from my previous observation during a ‘bed shortage’. (…)  I hear loud laughter coming from a patient’s room and see a group of five or six nurses inside, amusing themselves loudly about something. From the room comes the nurse who had looked after the patient in the morning and is now joining the handover. He immediately tells the late shift nurse that everyone is in a lighter mood today, which prompts him to add: “So don’t be surprised if I say something silly.”  *(Observation 5)* |
| **Bed shortage** | see article |
| **Continuity of care** |  |
| Continuity of clinicians in the care of a given patient appeared to shape decision work by influencing how well the patient was known. When knowledge of the patient’s course was available only through third parties, this affected how clinicians perceived the available options and positioned themselves in subsequent discussions. | In the next room lies an intubated but responsive patient. The consultant addresses the patient: “How are you feeling today? Do you feel strong enough to attempt an extubation?” The patient nods. The responsible nurse adds that she does not know the patient well herself but has heard from colleagues that the patient is apparently unable to swallow. *(Observation 9)* |
| **Team composition** |  |
| The situational constellation of clinicians, in interplay with individual and relational factors, appeared to influence decision work. For example, interprofessional collaboration was fostered when actors valued one another. | Looking at the group of physicians now entering the first room, led by the consultant, the nurse I am shadowing turns to me and remarks that “today they are all nice ones” – “no arrogance among them.” *(Observation 2)* |
| **Workload intensity** |  |
| High workload appeared to influence decision work, most visibly when lack of time hindered the execution of decisions. It also affected other aspects, for example when limited time reduced information gathering or when clinicians were too busy to take part. | While the bedside round, the consultant tells the responsible nurse that weaning from the ventilator should be pursued more consistently. The nurse addressed, however, is currently attending to the newly admitted patient in the same room and reacts rather sensitively, pointing out that she had no time for this. *(Observation 3)* |
| **System level; structural** | |
| **Guidelines** |  |
| Guidelines seemed to shape decision work, for example with regard to lung-protective ventilation, while their limitations were also acknowledged. | “But when it comes to how best to ventilate an intensive care patient, you can look at the guidelines and see that for many recommendations there is only very limited evidence. They often do not really state what is best, because ventilation is often something highly individual. Of course, I can specify six millilitres per kilogram, but what is the best PEEP for someone?” *(Interview 10, consultant, extensive ICU experience)* |
| **Laws/regulations** |  |
| Laws and regulations appeared to influence decision work, for example when nurses sought consultation with physicians for legal assurance. They also mattered in far-reaching ventilation decisions such as tracheotomies, particularly regarding who was legally entitled to decide when patients could not do so themselves—often in interaction with relatives. | “Legally, the final say lies quite clearly with the physician, or more precisely the consultant. Our new residents – or even those with, say, half a year of ICU experience – I have to inform and consult with them, of course, but I know full well that they cannot assess the situation any better than I can myself.” *(Interview 16, nurse, extensive ICU experience)* |
| **Research evidence** |  |
| Evidence on ventilation therapy sometimes explicitly entered clinical practice and could influence decision work. The inclusion of patients in studies, in turn, could shape decisions at the individual level, for example regarding whether to extubate first or to remove ECMO. | I ask the experienced nurse I am shadowing how he arrives at the settings for NIV. (…) He emphasises that what ultimately matters is keeping peak pressure permanently below 30, similar to invasive ventilation. He also refers to studies suggesting that persistently elevated ventilation pressures could lead to pulmonary fibrosis. *(Observation 14)* |
| **System level; situational** | |
| **Pandemic** | see article |
