# Supplementary material for: Contextualising ventilation decisions: an ethnographic study of factors shaping interprofessional decision-making
Source: BMC Nurs. 2026 Feb 7;25:237. doi: 10.1186/s12912-026-04381-w (PMC12994234; doi:10.1186/s12912-026-04381-w)
Supplement: Supplementary file 3 — Supplementary Material 3 [file 12912_2026_4381_MOESM3_ESM.docx]

**Supplementary Material 3: Links to empirical data**

| **Experience** | |
| --- | --- |
| **E1** | “Well, one has to say, in intensive care the nurses are at the bedside all the time and they actually do much more with the ventilation than we do. Unlike in the OR, for example, where we as anaesthetists are basically the ones who control everything. Here, I am much less familiar with the ventilators than with those in the OR. And accordingly, they would be the first point of contact, or often problems are solved without us, let’s say it like that. And if neither of us knows what to do, then you ask the respective supervisor. During the day we usually have consultants available, and at night there is always at least one specialist who can support.” (Interview 12, resident, early ICU experience)  “Well, then I first go to a colleague where I just know they also have a lot of experience, and then I discuss it with them. How do you see it? Do you have an idea what one could change? Because sometimes you just don’t get it yourself, or so, have a simple block or so, which thankfully doesn’t happen that often, but it can happen. (both laugh briefly) So, and then you do it together and, as I said, if all else fails, of course you go to the physician — but unfortunately they are not always experienced on every shift.” (Interview 15, nurse, mid-level ICU experience) |
| **E2** | “So, the autonomy, I would say, regarding extubation is such that we basically manage the sedation weaning and also the weaning on the ventilator quite independently. And it happens occasionally that during the midday round or in the morning review the ventilation mode is changed quickly without consulting the nurse. Like, ‘oh, he can breathe PSV.’ But then, if he happens to be awake, he takes two breaths, and later he is back to a frequency of five or six. Yes, that does happen and it also causes irritation. (both laugh) (…) And unfortunately, of course, it is like this: if you have your standing as an experienced staff member, then it happens less. It is with inexperienced staff that changes are not discussed or reported back, and then they are left wondering, like, ‘why is my patient on PSV and not breathing.’” (Interview 16, nurse, extensive ICU experience) |
| **E3** | An experienced nurse continues and says that despite the rather poor values he would consider an extubation. “There are always cases where you extubate against the values and it works,” he says. My impression is that, based on his experience and intuition, he believes this would also be the case with this patient. He tells me that the patient could in no way fulfil the weaning protocol. (Observation 9) |
| **E4** | During post-procedural care of a tracheotomised patient, an experienced ICU nurse recalled being told to continue sedation at the previous dosage, when ‘now the senior consultant came and said something different.’ He concluded matter-of-factly, ‘They do not always agree … but I just do as I want.’ He reduced the sedative rate from 20 to 15 ml/h while keeping opioid unchanged, adding, ‘For young, inexperienced colleagues, this is a real mess.’ (Observation 4). |
| **E5** | “Because, as I said, I find blood gas analysis in general as a diagnostic tool super, super important, because, as I said, you just quickly see changes that clinically you simply would not notice otherwise, and ultimately.” (Interview 7, resident, early ICU experience)  “Well, I am not one of those who come in and immediately do a BGA. Yes, there are people who say, I want that out of the way, I want it right away. I am a bit lazy in that regard. Not that I would not want to do a BGA, but I think, that is a blood draw, I discard something. I do not like to do that unnecessarily. And if I see that I have to adjust things anyway, then I adjust in the way that I think makes sense. I wait fifteen minutes, then I do my BGA and then I have a current picture. Of course, it is also very much dependent on experience. – There are automatisms which are certainly very valuable for someone less experienced. I do not want to deny that or speak badly of it, but it is important that before you can structure what is important and what is not important, you first do everything that could be important. That surely proves its worth as well.” (Interview 16, nurse, extensive ICU experience)  “Well, when I do get involved, it is often that it does not work out so well, and then, there is not one single parameter, but I look, for example, does the compliance get better, or can you somehow improve the peak, the pressure difference, with a higher PEEP, and such things. (...). So it is actually several parameters somehow, yes, but it is actually, so to speak, more objectifiable things on the monitor – and not somehow the gut feeling or so.” (Interview 17, resident, early ICU experience) |
| **E6** | “We as nurses of course do influence this quite significantly, because we also, I think, have a good standing here – and our professional experience. And I also have to say, the opinion of someone with a lot of professional experience and a specialist course and maybe other further training certainly carries much more weight than that of someone at the very beginning of their career.” (Interview 5, nurse, extensive ICU experience) |
| **E7** | “Of course, I cannot judge how it is when you have not been here long and have little experience yourself, and then also meet an inexperienced junior doctor. And that can, well, at least I experience it as charge nurse, that sometimes a colleague comes to me and says, ‘can you help me – I think the patient needs to be intubated, but the doctor does not see it that way.’ Then I let the colleague explain to me why they think so, and I can quite well represent that opinion to the doctor or the consultant. So I then stand next to my colleague and say, ‘well, colleague xy is at the bedside, and we are of the opinion that this patient needs to be intubated.’” (Interview 6, nurse, extensive ICU experience) |
| **‘Bed shortage’** | |
| **BS1** | “But yes, that is always a bit, on the one hand individual, and on the other, yes, sometimes it is simply also when you know you have bed pressure and, and, and, then you say, now it has to go quickly. Then of course you have to stay a bit realistic and put on the brakes and say, yes, but even so, we could by no means transfer, we also cannot in good conscience put the patient back to a non-ventilation bed, so it actually does not help us to force this now. Rather, have we already tried everything, do we have physio, do we have speech therapy already on board, can the patient actually do it? These are then the kinds of things that, yes, in a detailed round without time pressure, that works well.”  (Interview 16, nurse, extensive ICU experience) |
| **BS2** | “I attend a mortality and morbidity conference: at one point, a nurse voices her incomprehension that, in the case of a patient with an infaust prognosis, a final extubation had not been carried out earlier, even though there was severe bed and staff shortage.” (Observation 5) |
| **Rounds** | |
| **R1** | [At the bedside round]  We move on to the next room, and the seemingly young nurse follows, continuing to contribute very actively. He voices his assessment of the patient’s situation unsolicited, again positioning himself between the senior physician and the residents. In doing so, he offers his own perspective on the patient’s condition, which the physicians seem to acknowledge and take seriously. (Observation 4)  [At another bedside round]  The ventilator settings are also discussed. The consultant stands at the ventilator, looking at the monitor, and asks whether the low tidal volumes are intentional. One of the residents, who was already at the patient’s side and thus not part of the earlier rounds, explains that the tidal volume had been calculated that way because the patient is only 1.65 m tall. The consultant then checks the blood gas results on the computer, which reveal a marked acidosis under the current ventilator settings. He subsequently increases the inspiratory pressure.  Meanwhile, the nurse responsible for the patient remains in a relaxed position at the edge of the room and does not contribute to the discussion, though she appears to be attentively following the exchange. (Observation 4) |
| **R2** | [At the bedside weaning round]  We move on to the next room. The consultant, who had also presented the patients previously, again takes the lead. He outlines the situation, the course so far, and then – as seems to be the usual practice – passes the word to the physiotherapist.  The physiotherapist provides a brief but comprehensive assessment, describing relatively good responsiveness and motor reactions, and adding that the patient is able to make eye contact.  Next, as is customary, the occupational therapist speaks. She confirms all of this – except for the eye contact.  The speech and language therapist then follows, noting a good cough reflex, which, according to her, has also been observed by colleagues.  Afterwards, the nurse in the room is actively involved. On being asked, she confirms the observations regarding the patient’s cough ability, and adds: “Yes, the plan would be to get him out of bed into a chair.”  As during earlier parts of the round, everyone is once again gathered around the patient’s bed. The nurse responsible for organising the weaning round sits at the computer, takes notes, and remarks to the physicians: “The weaning plan is still missing here.” (observation 16) |
| **R3** | “Well, with some patients, yes, you get the feeling it’s mainly done to meet those extra reimbursement criteria. But with other patients I really do find it valuable – especially the active exchange with physio, speech therapy, and occupational therapy. Otherwise, you often just read two or three lines in the electronic documentation. But when everyone actually discusses things with each other, you get a much more comprehensive picture of the current situation and of what can be done to move the patient forward.” (Interview 13, resident, mid-level ICU experience)  [At the weaning visit, two consultants, two nurses, a physiotherapist, a speech therapist, an occupational therapist and a social worker gather around the patient.]  Another consultant enters the room. He is immediately confronted with the question of where he had been – posed by the senior consultant.  He replies curtly: “Well, I have to work.”  The remark hangs in the room for a moment.  The senior consultant responds: “Yes, this is work.”  Again, a pause follows.  Then the newly arrived consultant repeats: “Well, you can’t really get any proper work done during this time.” (Observation 16) |
| **Significant others** | |
| **SO1** | From the nursing side we had actually already expressed the wish that the handover round should, at least to a large extent, take place at the bedside, and that the physicians could also take the laptop with them to look at the x-ray or whatever else. But the consultant has a very firm opinion that this serves medical training, and that all media must be available there: one looks at the lab, one is for the x-rays, one for the orders. (Interview 16, nurse, extensive ICU experience) |
| **SO2** | “Well, we had a ventilation workshop at the beginning of the rotation, where there was a theory part and then, of course, things were shown to us on the machine. And then, before I came to the ICU, I also read a bit, well, about lung-protective ventilation and ventilation, and then from colleagues who had been there longer or from nurses — they also sometimes explained things about it.” (Interview 17, resident, early ICU experience)  Interviewer: “Thank you very much. What guides you then, when it comes to the settings — how do you know what exactly to set?”  Participant: “Well, what we of course have from anatomy and physiology, from the basic knowledge — and what makes a big difference, the onboarding, the two months that we had, which always takes place with colleagues who definitely have a specialist qualification.” (Interview 11, nurse, early ICU experience) |
| **SO3** | “In the past it was of course also like that, we had a great respiratory therapist who basically took everything in hand, and that is what we are missing now. And we notice that especially with the topic of ventilation. It leaves us extremely adrift. So our expertise has clearly become much weaker than ten years ago or so.” (Interview 5, nurse, extensive ICU experience)  So we have a colleague, especially [name] — you have already met him — he really is, when it comes to ventilation, the absolute crack. You can ask him about anything, he also does this simulator training, really, really great — honestly, top marks, that has to be said. You can always, always still learn something from him. I think at home he practically only reads ventilation books (laughs), or articles, or whatever, studies.” (Interview 2, nurse, extensive ICU experience) |
| **SO4** | “So one example comes to mind, there is this APRV ventilation. You probably know it too, right? That is a concept, and many in the US have actually been using it for many years and also successfully, and not only for patients with ARDS, but also others. But with us it only really came up with COVID, when [name of the nurse] simply thought about how these patients could be ventilated better, what could be done for them when they have such severe ARDS, and then he tried to introduce this mode of ventilation here.” (Interview 2, nurse, extensive ICU experience) |
| **Pandemic** | |
| **P1** | “If I now, for example, set a ventilation mode that the physician is not well trained in, or that I do not manage well myself — there are special ventilation modes where I lack the routine, because they only came up after I was away from the bedside. Of course under COVID it was all different again, but there, I often lack the routine.” (Interview 6, charge nurse, extensive ICU experience)  “When it comes to more difficult settings on the ventilator, somehow, that was especially during the COVID time, with patients who really hyperventilated so much on the ventilator, with massively high tidal volumes, where you then really sometimes almost had to relax them in order to prevent a SILI. That is then partly also difficult for us.” (Interview 13, resident, mid-level ICU experience) |
| **P2** | “Participant: Definitely. What really changed things a lot, I think, was COVID.  Interviewer: Oh, okay.  Participant: I believe most of us really made big progress in terms of ventilation, and also thought a lot about it, read a lot, learned a lot and so on. Because for a while we even had an extra folder where all the papers were collected, about COVID and then also more broadly about ARDS, ventilation regimes, ARDS and options — everything that came out in terms of studies. There was this extra folder in nursing, where sometimes even the consultants put things in, just so that people could read a bit and so on, and do a bit of further training.” (Interview 4, nurse, extensive ICU experience) |
